# Direct vs. Indirect Digital Implant Impressions: A Time and Cost Analysis

**DOI:** 10.3390/dj12110340

**Published:** 2024-10-25

**Authors:** Manuel António Sampaio-Fernandes, Ricardo Jorge Pinto, Paulo Rocha Almeida, Maria Margarida Sampaio-Fernandes, Duarte Nuno Silva Marques, Maria Helena Figueiral

**Affiliations:** 1 Faculdade de Medicina Dentária, Universidade do Porto, 4200-393 Oporto, Portugal; pralmeida@fmd.up.pt; 2 Faculdade de Medicina Dentária, Universidade de Lisboa, 1600-277 Lisbon, Portugal; ricardojpinto@edu.ulisboa.pt (R.J.P.); duarte.marques@campus.ul.pt (D.N.S.M.); 3 Faculdade de Medicina Dentária and INEGI Researcher, Universidade do Porto, 4200-393 Oporto, Portugal; mfernandes@fmd.up.pt

**Keywords:** cast scan, clinical efficiency, dental economics, dental scanning, digital dental impression, digital implant impression, digital workflow, time efficiency

## Abstract

Background/Objectives: This study aimed to compare the cost and time implications for implant-supported prosthesis comparing three digital impression techniques: digitization with an intraoral scanner, digitization of the conventional impression (without dental casts) and digitization of the stone models. Methods: To assess the time and cost of digital impression techniques on implants, time records on intraoral scans were consulted and three models were created with one, two and six implants to assess extraoral procedures time. Costs were evaluated based on material consumption, time expenditure and operator-related expenses. Time was recorded in three stages: (1) impression-taking, (2) model fabrication and (3) overall workflow completion. Statistical analysis was performed using ANOVA to compare cost and time differences across workflows. Results: Intraoral scanning presented a reduction in chair-side time between 4 and 20% when compared to extraoral techniques. For the three clinical situations evaluated, the intraoral scanning always presented the lowest cost. The extraoral digitization of impressions showed a reduction between 51.9% and 53.6% in laboratory time and between 3.5% and 7.6% in total cost compared to stone models digitization. Conclusions: The findings of this study indicate that intraoral scanning is a more cost-effective and time-efficient alternative to traditional impression methods, providing advantages in terms of reduced material use and shorter procedural durations.

## 1. Introduction

With technological advances in dentistry, the evaluation of various implant treatment protocols has become a topic of significant interest to both clinicians and dental technicians. Over time, surgical and prosthetic protocols evolved, leading to predictable treatments and high long-term survival rates for both implants and their prosthetic superstructures [1].

Computer-assisted design and computer-assisted manufacturing (CAD-CAM) technology has gained widespread acceptance in implant rehabilitation, demonstrating accuracy to conventional techniques for fixed prosthodontics [2]. Since traditional impression methods are prone to distortions and laboratory errors, which can contribute to misfits in implant-supported rehabilitations, using a digital workflow can improve efficiency, reducing chair time and omitting some laboratory steps [2,3,4].

Digital impressions can be captured using two primary methods: (i) Direct method—This involves using an intraoral scanner (IOS) to directly acquire a 3D virtual model without the need for conventional impressions; (ii) Indirect method—This method involves using an extraoral scanner (EOS) to scan conventional impressions or the stone models, which are then used to create a 3D virtual model [5,6,7,8]. Both methods produce standard tessellation language (STL) files, which are the three-dimensional dataset of the models. These files can be electronically stored and transmitted, thereby improving the overall efficiency of the workflow [9].

The digital workflow has been proposed as a potential method for reducing treatment costs [1]. However, studies that evaluate the economic parameters remain scarce in the dental literature [1,10]. Some studies estimated the economic efficiency in complete removable prostheses and others evaluated the cost differences between single-tooth replacement strategies [1,11,12]. Regarding implant-supported fixed prostheses, there is little scientific information [1]. Understanding the cost implications of different digital workflows is essential not only for clinicians, dental technicians and patients but also for health systems and society as a whole [13].

Although IOSs are increasingly used, they require a learning curve and involve a substantial initial investment [14]. Moreover, in certain clinical situations, the accuracy of IOS is reported to be lower than of extraoral scanning [15,16,17,18]. For these reasons, using extraoral scanners to digitize conventional impressions may serve as a viable alternative for implant rehabilitation [8,18].

In recent years, some studies have assessed the accuracy of various intraoral and extraoral scanners. However, few studies have focused on the accuracy of conventional impressions in conjunction with extraoral scanners [5,19,20,21]. Moreover, to the authors’ knowledge, no studies have thoroughly examined the time and cost associated with three different impression techniques: intraoral scanning, digitization of conventional impressions and digitization of stone models.

Given the range of digital impression techniques available, it is critical to understand their respective time and cost implications for different clinical scenarios. This information can aid in selecting the most appropriate technique for implant-supported reconstructions based on specific patient needs and practice constraints.

Therefore, the aim of the present in vitro study is to perform a cost and time analysis for implant-supported reconstructions by comparing three digital impression techniques: (A) digitization using an intraoral scanner, (B) digitization of the conventional impressions and (C) digitization of the stone models.

## 2. Materials and Methods

### 2.1. Acquisition of Reference Models

Three clinical scenarios were evaluated in this study: (i) single-unit implant-supported reconstruction, (ii) three-unit reconstruction supported by two implants and (iii) full-arch implant rehabilitation supported by six implants.

For each clinical scenario, three different techniques for obtaining working models were compared: (A) digitization in vivo using an IOS (TRIOS 4, 3Shape, Copenhagen, Denmark), (B) digitization in vitro of the conventional impression (without dental casts) and (C) digitization in vitro of the stone models.

A statistical power analysis was performed for sample size determination to obtain mean differences comparison between groups. With an α = 0.05, a power of 0.80, a standard deviation of 14 min and an effect size of 30, this analysis revealed that five samples per group would be needed.

For technique A, fifteen measurements were recorded (five for each clinical scenario). The intraoral scans were performed by seven postgraduate students with different degrees of experience with IOS.

**Figure 1:**
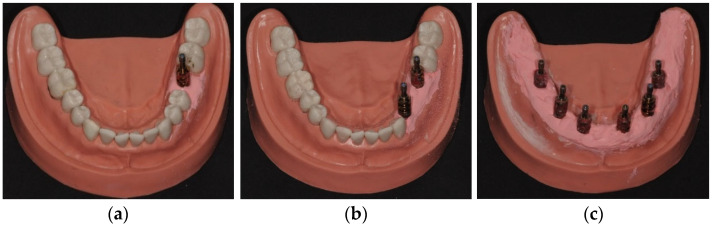
Models; (**a**)—M1; (**b**)—M2 and (**c**)—M3.

To evaluate the extraoral techniques (techniques B and C) in different clinical scenarios, three mandibular study models (KaVo Dental GmbH, Ulm, Germany) were used, each containing implants (Straumann bone-level 4.1 with internal hexagonal connection with multiunit): Model 1 (M1)—synthetic resin teeth and one implant at position 36 (Figure 1a); Model 2 (M2)—synthetic resin teeth with two implants at position 34 and 36, and a pontic at position 35 (Figure 1b) and Model 3 (M3)—six implants at positions 36, 34, 32, 42, 44 and 46 (Figure 1c). The materials and equipment needed to perform the different techniques were provided to the operator, who performed each measurement five times for each simulated clinical scenario. To standardize the procedures, each operator received eight hours of training, performing all scans according to the manufacturers’ manuals. Extraoral scans (techniques B and C) were performed using a blue LED scanner (Identica T500 Medit^®^, Seoul, Republic of Korea). After each scan, the scanners were turned off to allow a 10 min interval to cool the system before initiating a new scan sequence.

### 2.2. Intraoral Scanning

Intraoral scans (technique A) were performed with the 3Shape scanner (TRIOS 4) on a total of 15 patients at room temperature, with approximately 1000 lux of illuminance. Time measurements were recorded for the following steps: hardware startup, software setup, X-ray control, arch scanning with ScanBodies (CARES Mono ScanBodies, Straumann AG, Basel, Switzerland), antagonist scan, bite scan and data processing.

### 2.3. Extraoral Scanning

In the extraoral scans, material manipulation times followed the manufacturers’ recommendations. Time was recorded from the moment the elastomer mixing machine button was pressed until the end of the setting time was complete. Standardized plastic trays were selected according to the arch size and were drilled above the implant sites to allow a non-splinted open-tray impressions. Impression copings (Straumann AG, Basel, Switzerland) were torqued to 15 Ncm and radiographically verified for positioning accuracy. After confirming proper seating of the copings in the tray holes, impressions were taken with vinyl polysiloxane (VPS) (Light Body Type III, Putty Soft Type 0, Zhermack^®^, Rovigo, Italy) with a double-mixing technique. A silicone adhesive (Coltene^®^, Altstätten, Switzerland) was applied prior to elastomeric impression. For the antagonist arch, alginate impressions (Hydrogum 5, Zhermack^®^, Badia Polesine, Italy) in standard metallic trays were performed and the bite was registered with Occlufast Rock (Zhermack^®^, Badia Polesine, Italy). The working times provided by the manufacturers for adhesive application, elastomeric impression materials, antagonist arch impression materials, bite registration, dental stone and disinfectant use were considered.

### 2.4. Extraoral Scanning of Conventional Impressions

**Figure 2:**
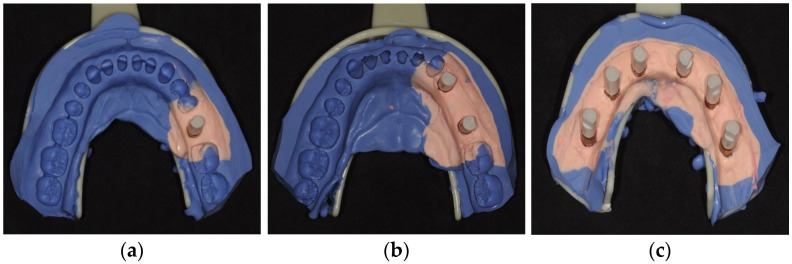
Impressions with ScanAnalogs; (**a**)—M1 impression; (**b**)—M2 impression and (**c**)—M3 impression.

In technique B (conventional impression scanning), the impressions were prepared by trimming excess material with a scalpel. ScanAnalogs (23.413.101.01-2 Dynamic Abutment, Lleida, Spain) were then screwed onto the impression copings, which were subsequently scanned using the extraoral scanner. Time measurements were recorded for the following steps: tray preparation, X-ray control, conventional impression, tray removal, impression preparation, ScanAnalogs placement (Figure 2), impression scanning and data processing.

### 2.5. Extraoral Scanning in Stone Models

In technique C (stone models scanning), after the conventional impressions, impression analogs (SRA 023.4756, Straumann AG, Basel, Switzerland) were screwed into the copings and the impressions were poured with type IV dental stone (Elite Rock REF: C410334 Zhermack^®^, Italy). The stone models were then scanned using ScanBodies (CARES Mono ScanBodies, Straumann AG, Basel, Switzerland). Times measurements were recorded for the following steps: tray preparation, X-ray control, conventional impression, tray removal, analog placement, pouring with gypsum, model preparation, ScanBodies placement, stone model scanning and data processing with the extraoral scanner.

### 2.6. Time Efficiency

To compare time efficiency across techniques and clinical situations, the time for each phase of the procedure was evaluated. The digital acquisition process was divided into three phases: preparation time (PT), working time (WT) and laboratory time (LT) in order to calculate the time spent in each phase (Table 1). Chair time (CT) was also calculated, which is the sum of PT and WT.

The total treatment time, representing the actual procedural time (excluding time allocated for case study preparation and office setup), was calculated for each technique. Impression material relaxation time and transport time to the laboratory were excluded, as they typically coincide, and transport time can vary based on the distance to the lab or the presence of an in-house laboratory.

For the purpose of comparison, the time elapsed from the start of the impression to the point when the laboratory received the STL file for digital design of the prosthetic component was recorded for each technique. Time for each step was measured using a stopwatch accurate to the nearest second.

### 2.7. Cost Efficiency

Cost efficiency was evaluated based on the materials used and the overall expenses incurred during impression acquisition.

The study assessed clinical treatment and laboratory fabrication costs across the three prosthetic workflows and a cost minimization analysis (CMA) was conducted based on the recorded treatment durations.

Prosthetic component costs were sourced from the same commercial provider whenever possible and were calculated based on single-use materials.

Capital investments for scanner acquisition, laboratory technician travel and software licensing fees were not included in the cost analysis.

To calculate the total cost of different techniques in an oral rehabilitation with implants, data from the Portuguese Dental Association (OMD) report titled “Determining the cost of treatments in dentistry” were used [22]. The reported costs correspond to a standard dental clinic with an office and dental assistant. Indirect costs, such as staff costs, equipment depreciation, miscellaneous instrument depreciation, office overhead and other costs (e.g., administrative expenses) were estimated at €89.18 per hour. Direct costs include the basic consultation kit, which was estimated at €4.78, and the additional costs of specific treatments [22].

## 3. Results

Mean times and 95% confidence intervals were recorded in Table 2 for the three clinical scenarios, the three digital impression techniques and the four treatment phases: preparation time (PT), working time (WT), chair-side time (CT) and laboratory time (LT).

Regarding the CT, techniques B and C present the same time indicators as they involve the same procedural steps in this phase. Technique A consistently demonstrated lower values compared to the extraoral techniques, with a 20% reduction in chair-side time observed for both the single-implant scenario (8:05 vs. 10:08) and the six-implant scenarios (12:32 vs. 15:27). However, in the two-implant scenario, the reduction was less pronounced, at only 4% (10:15 vs. 10:41).

Regarding LT, technique A does not involve any steps in this phase, as it is entirely digital. In contrast, technique B showed an average reduction of 53% in LT compared to technique C, reflecting the streamlined workflow of scanning the impression directly, rather than producing stone models.

The itemized costs of oral rehabilitation with one implant, two implants and six implants are recorded in Table 3. For rehabilitation with a single implant, technique A resulted in the lowest overall cost (€198.81), followed by technique B (€282.1) and technique C, which had the highest cost (€297.63). A similar trend was observed in the two-implant scenario, where technique A again presented the lowest costs (€523.29), while technique C was the most expensive (€724.11). In total rehabilitation involving six implants, technique A had the lowest cost (€1937.57), while technique C remained the highest at €2432.87. The difference in chair-side time between intraoral and extraoral techniques had an impact of 1.5% of the treatment cost, considering the cost-per-hour estimated used in this study.

The key differences between the three digital impression techniques are shown in Table 4.

## 4. Discussion

In this study, the time and costs associated with three different impression techniques (intraoral scanning, scanning of impressions and scanning of stone models) from the point of impression to the start of the CAD stage were recorded. This analysis was conducted in three distinct clinical situations involving implants. The results of this study demonstrated that intraoral scanning (IOS) required less clinical time, incurred lower associated costs and reduced laboratory steps compared to extraoral scanning techniques. Among the extraoral techniques, scanning of impressions showed a reduction in laboratory time and overall costs compared to stone model digitization.

In addition to clinical performance, the evaluation of economic parameters is crucial for decision-making in any treatment in general [23]. The cost minimization analysis performed in this study assumed that the three scanning techniques compared provided similar therapeutic outcomes and efficiencies. It is important to note that time and cost analyses for implant-supported oral rehabilitations are still rare in the scientific literature.

The conventional approach, which includes multiple laboratory and clinical steps, can be significantly reduced or even avoided with the use of digital scanning techniques. This reduction in both time and associated costs of the conventional process has been well documented in the literature [3,24,25].

However, while some studies have compared the time efficiency of intraoral scanning versus analogical techniques or the use of extraoral scanners on stone models, to the authors’ knowledge, this is the first time that the comparison between intraoral scanning, extraoral scanning of stone models and extraoral scanning of conventional impressions for implant-supported rehabilitations is performed [1,3,25,26,27].

One of the key advantages of intraoral digital impression techniques is the ability to correct errors without the need to repeat the entire procedure, resulting in a shorter working time compared to correcting errors in conventional techniques [25,28]. A 2021 review highlights that IOSs demonstrate high clinical and laboratory time efficiency in single implant cases, but that documentation supporting its use in multiple-implant workflows is lacking [20].

Several studies comparing conventional and digital impressions have consistently shown that the intraoral digital impressions are more time-efficient [1,18,24,25,27,28]. For example, an in vitro study by Patzelt et al. reported that IOS could save up to 23 min compared to conventional impressions [3].

A clinical study involving inexperienced operators showed that conventional impressions took on average 10 min, while IOS took around 4 min [27]. These values are lower than those observed in the present investigation, likely because the previous study aimed to obtain study models, a less complex procedure compared to impressions in oral rehabilitation. Similarly, Lee et al. found that chair-side time (CT) for conventional impressions for a single implant rehabilitation took 24.7 min, while IOS took 12.48 min [25]. These values, while higher than the present study’s findings for single implants, can be attributed to the inexperience of the operators.

A clinical study on single implants reported values of approximately 12 min in conventional impressions and 7 min in IOS [26]. This aligns more closely with the present study’s results. Across various studies, findings generally agree with the present study, though slight discrepancies in times may be due to operator experience, the use of different IOS models or additional time spent on rescans and retakes [25,26,27].

Joda et al. reported on a study involving patients selected for oral rehabilitation with two implant-supported single crowns with values of 33 min for the conventional chair-side technique and 189 min of laboratory time compared to 23.3 min and 158.1 min, respectively, for the IOS technique [1]. While this study evaluated the laboratory time through to the final rehabilitation, the present study only measured time up to the beginning of the CAD phase.

Cost analysis from the aforementioned study revealed that the total cost of the digital procedure was approximately 18% lower than the conventional procedure [1].

The present study corroborates this trend, showing that the IOS technique was be-tween 20.4% and 33.2% less expensive than extraoral scanning. Furthermore, scanning impressions were 3.5% to 7.6% cheaper than scanning stone models, primarily due to the elimination of the casting stage and implant analog costs. As the scanning impression technique becomes more widely adopted, the cost of ScanAnalogs is expected to decrease, which will likely increase the cost disparity between the two extraoral techniques. In the present study, the largest difference in chair time was observed in single implant rehabilitation scenarios, where the intraoral technique reduced chair time by 20% compared to the extraoral techniques. However, this time reduction contributed to less than 1.5% of the overall treatment cost, based on the hourly rate used in this study.

It is important to note that certain steps—such as applying adhesive before taking a conventional impression, disinfecting impressions prior to laboratory submission or initializing hardware and entering patient data—can affect the actual clinical time required. These steps may be performed before, during or after other procedures, which could alter the perceived efficiency. Additionally, many of these steps can be delegated to dental assistants, further optimizing the workflow [3].

Material handling times were based on the manufacturers’ guidelines, and the study did not account for the time required to remove temporary restorations or healing screws, as these steps are consistent across all techniques. Moreover, the time required for post-processing data, exporting files and importing them into CAD software (Exocad 3.1) was not considered in most studies. In situations where the data does not need to be exported from the IOS and subsequently imported into the CAD software, the results may be different [18]. In the present study, the time required for data processing, hardware initialization and software configuration were accounted for. While this study did not evaluate the investment and maintenance costs of equipment (such as intraoral scanners, extraoral scanners or elastomer mixing machines), it is important to recognize that these devices are commonly used across various dental areas such as orthodontics or conventional fixed prosthodontics. However, according to a survey carried out in 2023, the maintenance cost of the IOS varied considerably from a few hundred euros to more than 1000 euros per year in different countries. Factors such as frequency of use and the IOS brand are important considerations before purchasing a new IOS and software [29]. On the other hand, the transport time required in extraoral scanning techniques was not considered in this study and it represents one of the advantages of IOS techniques since the files are electronically sent to the laboratory.

One of the limitations of this study is having a small sample size. Increasing sample size would allow to obtain an intra subject comparison and not only the average times received from each group study.

Additionally, the learning curve associated with each technique should be considered when interpreting the results. However, a study by Róth et al. reported that, with Trios 4 IOS, fewer digital impressions were sufficient to reach the average scanning time of an experienced user compared with other IOSs [14]. It should also be noted that different implant systems offer varying workflow protocols, many of which are developed within closed systems, limiting comparability with other workflows. Large-scale clinical studies are needed to confirm the results of this investigation.

## 5. Conclusions

Intraoral scanning demonstrated a reduction in chair-side time between 4% and 20% compared to extraoral techniques in implant-supported rehabilitations. Moreover, intraoral scanning does not require any laboratory procedures prior to the CAD phase, streamlining the workflow. Among the extraoral techniques, the impression-scanning method reduced laboratory time by 51.9% to 53.6% compared to the scanning of stone models, highlighting its efficiency.

Based on the findings of this study, we concluded that each digital impression technique presented a distinct economic impact, due to differences in material usage. The reduction in time contributed to only a 2% difference in the overall cost. However, additional factors such as patient comfort and the accuracy of each digital workflow should also be considered in the decision-making process for selecting the appropriate technique.

## Figures and Tables

**Table 1:** Time treatment phases.

	TECHNIQUE A	TECHNIQUE B	TECHNIQUE C
PREPARATION TIME (PT)	Hardware startup	Impression coping placement	Impression coping placement
	Software setup	X-ray control	X-ray control
	ScanBodies placement	Tray selection	Tray selection
	X-ray control	Adhesive application	Adhesive application
WORKING TIME (WT)	ScanBodies scan	Conventional impression	Conventional impression
	Antagonist scan	Antagonist impression	Antagonist impression
	Bite scan	Bite registration	Bite registration
	Data Processing		
LABORATORY TIME (LT)		Washing and disinfection	Washing and disinfection
		Impression preparation	Analog placement
		ScanAnalogs placement	Poured with gypsum
		Impression scan	Model preparation
			ScanBodies placement
			Stone model scan

Technique A—Intraoral scanning; Technique B—Extraoral scanning of conventional impressions and Technique C—Extraoral scanning in stone models.

**Table 2:** Average times recorded at each stage for each clinical situation and each technique.

Clinical Situation	Technique	Preparation Time ^1^ (PT)	Working Time ^1^ (WT)	Chair-Side Time ^1^ (CT)	Laboratory Time ^1^ (LT)	Summary Time
1 Implant	A	2:23 [2:17; 2:29]	5:43 [5:03; 6:23]	8:05 [7:21; 8:50]	Not applicable	8:05 [7:21; 8:50]
	B	1:36 [1:32; 1:40]	8:32 [8:25; 8:39]	10:08 [10:03; 10:13]	14:08 [14:04; 14:11]	24:16 [24:07; 24:24]
	C				30:29 [30:24; 30:33]	40:37 [40:27; 24:46]
2 Implants	A	2:34 [2:15; 2:54]	7:40 [2:46; 12:34]	10:15 [5:29; 15:01]	Not applicable	10:15 [5:29; 15:01]
	B	1:51 [1:45; 1:57]	8:51 [8:33; 9:08]	10:41 [10:28; 10:54]	14:30 [14:20; 14;40]	25:11 [24:48; 25:36]
	C				30:46 [30:44; 30:48]	41:27 [41:12; 41:42]
6 Implants	A	3:43 [3:06; 4:19]	8:49 [3:57; 13:41]	12:32 [7:47; 17:18]	Not applicable	12:32 [7:47; 17:18]
	B	3:53 [3:39; 4:04]	11:34 [11:18; 11:49]	15:27 [15:13; 15:41]	15:46 [15:26; 16:06]	31:13 [30:39; 31:47]
	C				32:46 [32:01; 33:31]	48:13 [47:14; 49:12]

^1^ (minutes:seconds); Technique A—Intraoral scanning; Technique B—Extraoral scanning of conventional impressions; Technique C—Extraoral scanning in stone models.

**Table 3:** Itemized cost of oral rehabilitation with implants in a standard clinic.

Technique	Technique Treatment Costs (in Euros)		1 Implant	2 Implants	6 Implants
A Intraoral Scanning	Indirect Costs (89.18 × time in hours)		12.01	15.23	18.63
	Direct Costs	Basic kit (4.78/unit)	9.57	14.36	19.14
		ScanBodies (5.1/unit)	5.1	10.2	30.06
		Composit	0.27	0.54	1.62
		Articulating paper (80, 40, 8 micra)	0.77	0.77	1.52
		Laboratory invoice	171.09	482.19	1866.6
		Total	186.8	508.06	1918.94
	TOTAL		198.81	523.29	1937.57
B Conventional impression scanning	Indirect Costs (89.18 × time in hours)		15.06	15.88	22.96
	Direct Costs	Basic kit (4.78/unit)	9.57	14.36	19.14
		Impression coping (41/unit)	41	82	246
		Silicone Putty 26 mL	3.81	3.81	3.81
		Silicone light 10 mL	6.57	6.57	6.57
		Alginate (20 g)	0.6	0.6	0.6
		Registration bite	4.36	4.36	4.36
		ScanAnalogs (29/unit)	29	58	174
		Composit	0.27	0.54	1.62
		Articulating paper (80, 40, 8 micra)	0.77	0.77	1.52
		Laboratory invoice	171.09	482.19	1866.6
		Total	267.04	653.2	2324.22
	TOTAL		282.1	669.08	2347.18
C Stone model scanning	Indirect Costs (89.18 × time in hours)		15.06	15.88	22.96
	Direct Costs	Basic kit (4.78/unit)	9.57	14.36	19.14
		Impression coping (41/unit)	41	82	246
		Silicone Putty 26 mL	3.81	3.81	3.81
		Silicone light 10 mL	6.57	6.57	6.57
		Alginate (20 g)	0.6	0.6	0.6
		Registration bite	4.36	4.36	4.36
		Stone models	39.43	39.43	39.43
		Implant analog (31.7/unit)	31.70	63.4	190.2
		ScanBodies (5.1/unit)	5.1	10.2	30.06
		Composit	0.27	0.54	1.62
		Articulating paper (80, 40, 8 micra)	0.77	0.77	1.52
		Laboratory invoice	171.09	482.19	1866.6
		Total	282.57	708.23	2409.91
	TOTAL		297.63	724.11	2432.87

Indirect costs based on data obtained from Table 2.

**Table 4:** Differences between digital acquisition techniques.

	Intraoral Scanning	Conventional Impression Scanning	Stone Model Scanning
Physical Impression	No	Yes	Yes
Intraoral Cam	Yes	No	No
Stone model	No	No	Yes
ScanBodies	Yes	No	Yes
ScanAnalogs	No	Yes	No
Cost	Average	High ^1^	High ^1^
Clinical chair time	Average	High	High
Laboratory work time	Not applicable	Average	High

^1^ cost difference between extraoral techniques less than 5%.

## Data Availability

The original contributions presented in this study are included in the article.
